# Placeboeffekte bei der Therapie mit Antidepressiva: Implikationen für die wissenschaftliche Bewertung und klinische Anwendung medikamentöser Depressionsbehandlungen

**DOI:** 10.1007/s00115-024-01784-5

**Published:** 2024-12-12

**Authors:** Winfried Rief, Tobias Kube

**Affiliations:** 1 Klinische Psychologie und Psychotherapie, Philipps-Universität Marburg, Gutenbergstraße 18, 35032 Marburg, Deutschland; 2 Klinische Psychologie und Psychotherapie, Rheinland-Pfälzische Technische Universität Kaiserslautern-Landau, Landau, Deutschland; 3 Klinische Psychologie und Psychotherapie, Georg-August-Universität Göttingen, Göttingen, Deutschland

**Keywords:** Placebo, Nocebo, Studiendesign, Erwartungen, Shared decision making, Placebo, Nocebo, Study design, Expectations, Shared decision making

## Abstract

**Hintergrund:**

Verblindete Placebobehandlungen in klinischen Studien erreichen oft bis zu 80 % der klinischen Verbesserungen der Verumgruppen. Offensichtlich ist der größte Teil der Wirkung, die in klinischen Studien in den Antidepressivagruppen gefunden wird, auf Faktoren zurückführen, die nicht spezifisch für die Antidepressivatherapie sind. Im Artikel wird dargelegt, welche Faktoren zu dieser hohen Effektivität von Placebointerventionen bei Antidepressiva beitragen.

**Methodik:**

Es wird ein narrativer Literaturüberblick mit besonderer Berücksichtigung der Placeboeffekte in Studien zu Antidepressiva vorgestellt.

**Ergebnisse:**

Bei Depressionen findet sich auch im Vergleich zu anderen psychischen Störungen ein besonders starker Placeboeffekt. Die Stärke dieses Effekts lässt sich modulieren durch hilfreiche vs. nichthilfreiche Instruktionen, durch Beteiligung der Patienten am Entscheidungsprozess, aber auch durch persönliche Zuwendung, insbesondere wenn die Behandler als kompetent und warmherzig wahrgenommen werden. Auch das Auftreten dezenter Nebenwirkungen kann Placeboeffekte verstärken. Placeboeffekte zeigen sich dabei nicht nur in subjektiven Patientenberichten, sondern auch durch nachweisbare neurochemische Veränderungen im Körper.

**Schlussfolgerung:**

Behandler tragen durch ihr eigenes Verhalten wesentlich zur Effektivität antidepressiver Behandlungen bei. Gleichzeitig muss der insgesamt doch eher geringe Abstand zwischen Verum- und Placebobehandlungen bei Depressionen eine kritische Beleuchtung der Bewertung des Kosten-Nutzen-Verhältnisses am Einzelfall nach sich ziehen. Studiendesigns zur Evaluation von Antidepressiva sind gefordert, die die komplexen Wechselwirkungen zwischen der eigentlichen Medikamentenwirkung und Placeboeffekten besser darstellen.

## Abstract (en)

**Background:**

Blinded placebo treatment arms in clinical trials often achieve up to 80% of the clinical improvements of the verum groups. Apparently, the majority of the effects found in the antidepressant groups in clinical trials are due to factors that are not specific to antidepressant treatment. This article reviews the factors that contribute to the high effectiveness of placebo interventions for antidepressants.

**Methods:**

A narrative literature review is presented with particular emphasis on placebo effects in antidepressant clinical trials.

**Results:**

There is a particularly strong placebo effect in depression compared to other mental disorders. The magnitude of this effect can be modulated by helpful versus nonhelpful instructions, by patient participation in the decision-making process but also by personal attention, especially if the clinician is perceived as competent and warmhearted. The occurrence of subtle side effects can also reinforce placebo effects. Placebo effects are not only reflected in subjective patient-reported outcome variables but are also evident in neurochemical changes in the body.

**Conclusion:**

Clinicians essentially contribute to the effectiveness of antidepressant treatment through their own behavior. At the same time, the relatively small overall difference between verum and placebo treatments for depression leads to the necessity of a critical evaluation of the cost-benefit ratio, adapted to the individual case. Study designs for the evaluation of antidepressants are required that better reflect the complex interactions between the genuine drug effects and placebo effects.

## Hintergrund

Kliniker möchten ihren Patienten effektive Behandlungsangebote machen können und Wissenschaftler sind in der weit überwiegenden Mehrzahl daran interessiert, durch Studien die Wirksamkeit bestimmter Behandlungsmaßnahmen nachzuweisen. Nicht nur im Bereich der Psychiatrie, sondern in der gesamten Gesundheitsversorgung führt dies zu einem gewissen „Effektivitätsbias“: Es gibt eine Tendenz, die positiven Effekte von Therapiestudien stärker darzustellen als Negativeffekte. In früheren Jahren führte dies auch zu einer stark selektiven Publikationsstrategie, sodass Studien mit positiven Ergebnissen häufiger publiziert wurden als Studien mit negativen Ergebnissen [37]. Die früher oftmals euphorische Darstellung der Möglichkeiten durch Psychopharmaka – aber auch durch Psychotherapie – weicht aktuell einer sachlicheren, selbstkritischen, oftmals auch desillusionierenden Perspektive, die mit einer deutlichen Reduktion der erwarteten spezifischen Effekte von Behandlungen einhergeht. Psychopharmakologische Weiterentwicklungen kommen nur schleppend voran, und es wird die Vermutung geäußert, dass Placeboeffekte bei psychischen Erkrankungen, insbesondere bei Depressionen, oftmals zu stark sind, um spezifische Medikamenteneffekte im Kontrast dazu darstellen zu können [13].

Der nachfolgende Artikel greift die Frage auf, wie stark Placeboeffekte bei Antidepressivastudien sind, wodurch sie moduliert werden und welche Implikationen dies für Behandler haben kann. Placeboeffekte werden dabei nicht nur als Bedrohung von Wirkungsnachweisen gesehen, sondern auch als Leitfaden für Behandler, erfolgreiche Behandlungen durchzuführen.

## Wie groß ist der wirkliche Vorteil von Antidepressiva über Placebo?

Als richtungsweisend für die Bewertung von Antidepressiva gilt die Arbeit von Cipriani et al. [3]. In einem besonders sorgfältigen Vorgehen, das auch unpublizierte Studien berücksichtigte, wird von einem Vorteil von Antidepressiva über Placebo in Höhe einer Effektstärke von SMD(standardisierte Mittelwertdifferenz)/d = 0,30 ausgegangen. Auch wenn dieser Vorteil als robust bewertet wurde, gilt er entsprechend den üblichen Konventionen als kleiner Effekt mit fraglicher klinischer Relevanz. Wie fragil der spezifische Wirkungsnachweis für Antidepressiva ist, zeigt sich auch aus Analysen, die auf Daten der amerikanischen Zulassungsbehörde Food and Drug Administration (FDA) aufbauen und zum Schluss kommen, dass jede zweite Antidepressivastudie nicht in der Lage ist, überhaupt einen systematischen Vorteil für ein Verum nachzuweisen [13, 14]; ein spezifischer Vorteil von Antidepressiva wird hier mit einem Hamilton-Score von HAMD = 2,5 ausgewiesen, was niedriger ist als der festgelegte Wert für einen klinisch relevanten Vorteil (üblicherweise > 4–6 Punkte Reduktion). Je nach Gruppe der Antidepressiva erreichen die Placebogruppen zwischen 71 % und 81 % der Effektivität der Antidepressivagruppen [15].

Detailliertere Informationen finden sich in einer neuen groß angelegten Metaanalyse, in die Daten von 57.300 Patienten mit schweren Depressionen eingingen [22]. Auch hier wurden FDA-gemeldete, jedoch ggf. nicht publizierte Studien berücksichtigt. Vergleichbar zu den Khan-Studien erreichte weniger als die Hälfte der teilnehmenden Patienten ein entsprechendes Responsekriterium (44,7 %), während in der Placebogruppe 35,8 % dieses Kriterium erreichten (somit 81 % der Verumgruppe). Zusätzlich wurde analysiert, ob die Placeboresponder ggf. diejenigen sind, die nur kleinere Vorteile entwickeln, während die wirklich großen Vorteile nur auf die Medikamentengruppe zurückgehen sollten. Dies konnte in sehr detaillierten Analysen jedoch nicht nennenswert den starken Effekt in den Placebogruppen erklären.

Grundsätzlich ist darauf hinzuweisen, dass diese starken Effekte in den Placebogruppen nicht zwingend auf spezifische Placebomechanismen zurückzuführen sind, sondern auch methodische Aspekte zu diesen Effekten beitragen. Die sog. „Regression zur Mitte“ als statistischer Effekt bzw. „episodischer Verlauf“ als klinischer Effekt tragen ebenfalls zu Verbesserungen im klinischen Status der teilnehmenden Patienten bei und können substanzielle Anteile dieser Unterschiede erklären (siehe z. B. [4]). Auf der anderen Seite stehen Studien, die zeigen, dass in Patientengruppen ohne jegliche Medikation die Effekte deutlich niedriger sind als in Behandlungsgruppen mit Placebomedikation [18]. Dies impliziert, dass der größere Anteil der Vorteile in Placebogruppen auf spezifische Placebomechanismen wie Erwartungseffekte, assoziatives Lernen wie klassische Konditionierung und Interaktion mit dem Behandler zurückgeht (für eine ausführlichere Diskussion dieser Effekte s. unten) und nicht auf statistisch-methodische Effekte.

Um diese Ergebnisse in positiveres Licht zu stellen, wird von einigen Autoren darauf hingewiesen, dass ggf. auch bei anderen medizinischen Maßnahmen nur niedrige spezifische Effektstärken gefunden werden [17]. Allerdings sind solche Vergleiche nicht belastbar. Effektstärken hängen naturgemäß von der Art der Variablen ab, und eine Effektstärke zur Verhinderung der diabetischen Retinopathie bedeutet z. B. etwas anderes als eine Effektstärke zum subjektiven Wohlbefinden. So ist sogar innerhalb des Feldes der Depressionsbehandlung die Abhängigkeit von der Zielvariablen belegt: Prä-post-Effektstärken sind am höchsten, wenn Expertenratings verwendet werden (also z. B. Hamilton-Depressionsskala; Montgomery-Asperg-Rating-Skala; [32]); bei subjektiven Einschätzungen von Patienten (z. B. Beck-Depressionsskala) finden sich geringere Effekte. Für die Bewertung von Depressionsstudien ist dies von besonderer Relevanz, da gerade bei Antidepressiva die überwiegende Mehrzahl der Studien Expertenratings verwendet.

**Abb. 1:**
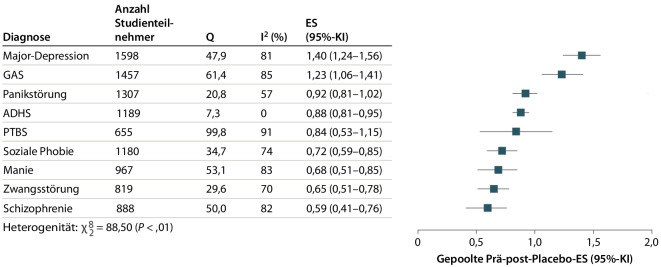
Placeboeffekte in den verschiedenen psychischen Störungsgruppen. *ADHS* Aufmerksamkeitsdefizit-Hyperaktivitäts-Störung, *GAS* generalisierte Angststörung, *ES* Effektstärke, *KI* Konfidenzintervall, *PTBS* posttraumatische Belastungsstörung

Allerdings scheint bei kaum einer Patientengruppe der Placeboeffekt so stark ausgeprägt zu sein wie bei Patienten mit Depressionen. Neueste Analysen der Effekte in den Placeboarmen weisen zumindest sehr deutlich darauf hin (Abb. 1; aus [2]).

## Wirken Placebos auch, wenn sie offen verabreicht werden?

Seit einigen Jahren gibt es intensive wissenschaftliche Diskussionen dazu, ob Placebos auch dann depressive Symptome reduzieren könnten, wenn sie mit voller Offenheit und Transparenz verabreicht werden, sog. Open-label-Placebos (OLP). Eine klinische Studie mit einer kleinen Stichprobe erbrachte vielversprechende Hinweise für die Wirksamkeit von OLP zur Reduktion depressiver Symptome bei nichtgeriatrischen Patienten über einen Zeitraum von 4 Wochen im Vergleich zu „treatment as usual“ [23]. Auch in experimentellen Studien zeigte sich, dass OLP Menschen mit Depressionen vor dem Erleben intensiver Traurigkeit schützen können [10]. Allerdings mehren sich die Befunde, dass OLP im Vergleich zu klassischen Placebos, die als potente Antidepressiva angekündigt werden, weniger wirksam sind [9, 10].

## Nebenwirkungen von Antidepressiva und Noceboeffekte

Berichte über Nebenwirkungen sind sehr stark von subjektiven Einschätzungen und Erwartungen abhängig. Am Beispiel von Venlafaxin konnte anhand landesweiter Beobachtungen zu Nebenwirkungsberichten in Neuseeland festgestellt werden, dass ungünstige Berichte in Massenmedien über das Medikament dazu führten, dass im Gefolge auch deutlich mehr Nebenwirkungen berichtet werden, und zwar zeitkontingent zu negativen öffentlichen Berichterstattungen [20]. In eigenen Analysen konnte bestätigt werden, dass auch in Placebogruppen von Antidepressiva primär jene Symptome berichtet wurden, die als potenzielle Nebenwirkungen an Patienten bei der Patientenaufklärung zuvor eingeführt worden waren (z. B. Mundtrockenheit bei Placebos in Trizyklikastudien [34]). Nebenwirkungen können somit durch ungünstige Instruktionen der Behandler verstärkt werden.

Allerdings sind Nebenwirkungen auch durch frühere Behandlungserfahrungen gebahnt. In einer experimentellen Studie mit Amitriptylin konnte gezeigt werden, dass auch bei Gesunden eine viertägige Amitriptylingabe zur Nacht dazu führt, dass wenige Tage später auf die Gabe von Placebo bereits Nebenwirkungen berichtet werden [28]. Dies unterstreicht, dass Patienten lernen können, auf Medikamente mit Nebenwirkungen zu reagieren. Diese Lerneffekte sind oftmals weniger an einzelne Medikamentengruppen gebunden, was erklärt, warum Patienten auch bei einem Wechsel zwischen pharmakokinetisch unterschiedlichen Präparaten trotzdem weiterhin Nebenwirkungen berichten können.

## Was beeinflusst Medikamenten- und Placeboeffekte?

Ob und wie stark Psychopharmaka wirken, hängt wesentlich von der Instruktion des Behandlers beim Verordnen der Medikamente ab. In mehreren experimentellen Studien konnte gezeigt werden, dass allein die Gabe eines Placebonasensprays (Cover Story: Testung einer neuen Applikationsform von Citalopram) dazu führt, dass sowohl gesunde Probanden als auch Patienten mit dem Vollbild einer Depression deutlich weniger Traurigkeit bei entsprechenden traurigstimmenden Stimuli entwickeln, aber auch weniger Rumination und andere depressionsspezifische Merkmale, als wenn dasselbe Placebo mit einer anderen Instruktion gegeben wird [10, 27].

Besonders eindrücklich konnte dieser Effekt unter Verwendung eines realen Antidepressivums (Escitalopram) gezeigt werden. Dieses Medikament wurde bei sozial ängstlichen Personen verabreicht, also in einem Indikationsbereich, für den ein Wirkungsnachweis vorliegt. Allerdings zeigen sich die positiven Wirkungen nur, wenn der Behandler das Medikament mit der Instruktion einführt, dass es sich hier um eine nachgewiesene effektive Behandlung handeln würde. Wird das Medikament bei sozial ängstlichen Personen verabreicht, jedoch mit der Instruktion, es handelt sich um ein Placebo, das nur aus wissenschaftlichen Gründen als Vergleichsbedingung eingesetzt wird, bricht die Effektivität dramatisch auf weniger als ein Drittel ein [8]. Dies ist ein eindrücklicher Beleg, dass Antidepressiva nur dann ihr volles Wirkungspotential entfalten, wenn die Instruktionen auch entsprechend konstruktiv sind.

Aber auch andere Effekte tragen zur Wirkungsweise von Antidepressiva und anderen Medikamenten bei. Werden Patienten am Entscheidungsprozess für Medikamente beteiligt („shared decision making“), entwickeln diese Medikamente weniger Nebenwirkungen [1]. Eine besondere Rolle spielt auch die persönliche Zuwendung durch den Behandler. Ist in Studien viel persönliche Zuwendung durch Studienärzte oder „study nurses“ vorgesehen, erreichen die Placeboarme eine ähnliche Effektivität wie die Medikamentenarme [35]. Kaptchuk et al. [12] konnten bei Personen mit Reizdarmsyndrom zeigen, dass ein emotional zugewandter, emphatischer Behandler deutlich höhere Effektstärken in der Behandlung erreichen kann als ein wortkarger, sehr technisch wirkender Behandler.

In eigenen Studien konnte die Rolle subjektiver Behandlervariablen im Kontext von Psychotherapie weiter präzisiert werden, indem auf das sozial-psychologische Modell zurückgegriffen wurde, nach dem bei der sozialen Wahrnehmung primär eine Einschätzung auf den Dimensionen „Kompetenz“ und „Wärme“ erfolgt. Wenn Behandler mehr von diesen Variablen Wärme und Kompetenz zeigen, sind sie überzeugender und stimulieren positivere Behandlungserwartungen bei Patienten [36]. Die Dimensionen Wärme und Kompetenz tragen entsprechend auch zu stärkeren Placeboeffekten bei und stellen einen Puffer vor der Entwicklung von Nebenwirkungen dar [11].

Zudem gibt es Hinweise darauf, dass die Art und Weise, wie die Wahrscheinlichkeit des Auftretens gewünschter (z. B. Symptomreduktion) und unerwünschter Wirkungen (z. B. Nebenwirkungen) von Behandlern kommuniziert wird, das Symptomerleben beeinflusst: So wird im Kontext von Influenzaimpfungen eine geringere Belastung durch Nebenwirkungen erlebt, wenn der Behandler über eine hohe Wahrscheinlichkeit für das Nichtauftreten von Nebenwirkungen (z. B. 90 %) aufklärt, anstatt die niedrige Wahrscheinlichkeit für das Auftreten (z. B. 10 %) zu kommunizieren [24]. Moderate Nebenwirkungen können z. T. Placeboeffekte verstärken und eine Bedrohung der Verblindung in klinischen Studien darstellen, wie Belege aus dem Schmerzbereich zeigen [31].

## Placeboeffekte bei Depressionen: Just in mind?

Zwar zeigen sich Placeboeffekte besonders stark bei subjektiven Variablen, jedoch ist die Aussage, dass Placeboeffekte deshalb primär subjektiv seien, klar zurückzuweisen. In vielen Bereichen konnten Placeboeffekte auch bei biologischen Parametern gezeigt werden [26]. Besonders beeindruckend sind hierbei sicherlich Studien von Eippert et al. [6], die aufzeigen, dass bei peripher applizierten Schmerzreizen bereits auf Höhe des Rückenmarks unterschiedliche neuronale Aktivierungsprozesse auftreten, je nachdem, welche verbalen (Placebo‑)Instruktionen die teilnehmenden Personen bekamen. Auch im affektiven System lassen sich insbesondere durch die funktionelle Bildgebung neurobiologische Prozesse darstellen, die je nach Intensität der Placeboreaktion unterschiedlich sind [25].

## Die neuen Hoffnungen der pharmakologischen Depressionstherapie

Gerade vor dem Hintergrund der zunehmend kritischeren Evaluation der Effektivität von bisher zugelassenen Antidepressiva werden neue Möglichkeiten gesucht. Kandidaten hierfür sind z. B. Ketamin/Esketamin oder Psilocybin. Vor dem Hintergrund, dass bei Medikamenten wie Ketamin Erwartungseffekte schwer auszuschließen sind, da Patienten die psychotrope Wirkung des Medikaments spüren, hat eine Studie von Lii und anderen [19] großes Aufsehen erregt. Um die Erwartungseffekte auszuschalten, wurden nur Patienten mit Depression in die Studie aufgenommen, die sich aus orthopädischen Gründen einer Operation unterziehen mussten. Die Hälfte der Patienten erhielt während der Operation/Narkose zusätzlich Ketamin verabreicht, die andere Hälfte Placebo. Sind so Erwartungseffekte komplett ausgeschaltet, zeigt sich kein echter Vorteil für die Behandlung mit Ketamin. Dem gegenüber zeigen sich deutliche Unterschiede in Abhängigkeit davon, ob Patienten glauben, Ketamin bekommen zu haben (unabhängig davon, ob sie wirklich Ketamin bekamen oder nicht).

Auch geht mit diesen Behandlungen in aller Regel eine starke persönliche Zuwendung einher sowie Behandlungssettings mit vielen positiven Hinweisreizen, die ein psychodelisches Erleben verstärken können. Trotzdem gibt es auch hier bereits erste Metaanalysen zum Vergleich der Effektivität in Placeboarmen vs. Verumarmen, z. B. beim Ketamin und Esketamin [21]. Bei 14 publizierten Studien konnten bereits im Placeboarm im Durchschnitt 72 % der Effektivität des Verumarms erreicht werden. Auch wenn dies erste Hinweise für eine starke Placebobeteiligung sind, so kann diese Bewertung noch nicht als abschließend betrachtet werden, zum einen wegen der o. g. Schwierigkeiten einer echten Verblindung und Ausschaltung von Erwartungseffekten, zum anderen auch wegen der noch nicht ausreichenden Berücksichtigung nicht publizierter Studien.

## Schlussfolgerungen für Wissenschaft und Klinik

Der größte Anteil der in Studien gefundenen Wirkung von Antidepressiva ist nicht spezifisch für die Pharmakodynamik des Präparats, sondern durch zahlreiche andere Faktoren (mit-)beeinflusst. Dies bringt einerseits neue Herausforderungen, aber andererseits auch neue Chancen. Gerade für den Behandler betont es letztendlich die Relevanz, mit welchen Instruktionen und welchem Interaktionsverhalten Medikamente verabreicht werden. Selbst bei so massiven Eingriffen wie in der Herzchirurgie konnte zwischenzeitlich gezeigt werden, dass die Optimierung von Patientenerwartungen an die Behandlung die Behandlungseffektivität steigern kann [33]. Dabei geht es nicht um übertrieben positive, sondern realistische Vorankündigungen, die für die Wahrnehmung positiver Behandlungseffekte sensibilisieren. Die Information über mögliche Negativeffekte muss weiterhin erfolgen, sollte jedoch balanciert eingebunden sein in die Gesamtinformation. Durch eine starke therapeutische Beziehung, die durch die Wahrnehmung von Kompetenz und Wärme auf Seiten der Behandler geprägt sein sollte, können insbesondere auch Negativeffekte reduziert werden (Infobox 1).

Trotzdem muss auch das Kosten-Nutzen-Verhältnis der Antidepressivagabe neu bewertet werden und fordert eine Entscheidung im Einzelfall. Behandler müssen sich bewusst sein, dass viele unserer bisherigen Ergebnisse einen starken positiven Bias zugunsten einer medikamentösen Therapie haben, gleichzeitig auch die Erfassung von Negativeffekten in verschiedenen Bereichen der Medizin eher stiefmütterlich erfolgte (siehe z. B. [29]). Da im Rahmen von Zulassungsstudien längere Beobachtungen nicht relevant sind, führen weit über 90 % der Medikamentenstudien keine Analyse von Langzeiteffekten durch. Entsprechend gering belastbar sind unsere Erkenntnisse zu möglichen Vorteilen einer Langzeitbehandlung mit Antidepressiva [5]. Gegebenenfalls ist jedoch bereits die Frage nach der spezifischen Effektivität von Antidepressiva falsch gestellt: Da es sich bei Depressionen um eine biopsychosoziale Erkrankung handelt, müsste deshalb ggf. von vorneherein die Frage so gestellt werden, ob und wann in Interaktion mit welchen Umgebungsbedingungen Antidepressiva hilfreich sind und bessere Effekte haben, als einfach nur Placebointerventionen durchzuführen bzw. Verhaltensempfehlungen zu geben. Eine Reformulierung der Wirkung von Antidepressiva als potenzielle „Verstärker“ anderer positiver Einflüsse auf betroffene Patienten könnte zu neuen Perspektiven und Möglichkeiten führen [30].

Grundsätzlich gilt jedoch prinzipiell unter der Placeboperspektive, dass typische Therapiestudien ein großes Manko mit sich bringen, wenn sie dem klassischen einfachen zweiarmigen Design folgen, das verblindet Verum mit Placebobedingungen vergleicht. Dabei besteht keinerlei Chance, Interaktionen zwischen den studienimmanent realisierten Placebomechanismen von Behandlungsbedingungen mit dem spezifischen Wirkstoff zu analysieren. Da Pharmafirmen in der Festlegung des Studiendesigns automatisch auch eine Festlegung möglicher Placebomechanismen vornehmen, die üblicherweise nicht weiter variiert wird, befindet man sich im Blindflug, ob die im Studiendesign realisierten Placebomechanismen in einem Bereich sind, die die Entdeckung spezifischer Verumeffekte erleichtert oder erschwert. Die früher postulierte Annahme, dass grundsätzlich Placebomechanismen reduziert werden sollen, um Verummechanismen aufzuzeigen, ist wissenschaftlich zu hinterfragen [16]. Manche Medikamente benötigen gute „Placebomechanismen“, um ihr Potenzial im vollen Umfang zu zeigen. Dies kann nur mit neuen Studiendesigns, z. B. „balanced placebo designs“, d. h. einer Variation der Substanz (Placebo vs. Verum) und Instruktion („Sie erhalten ein Placebo“ vs. „Sie erhalten ein wirksames Medikament“; [7]) im Sinne eines 2 × 2-Designs entdeckt werden.

### Infobox 1 Implikationen für Behandler, die Psychopharmaka verschreiben

1. *Patientenerwartungen an Behandlung erfragen und ggf. darauf eingehen*
  Nur wenn man Patienten nach ihren Erwartungen fragt, kann man auch feststellen, ob diese dysfunktional sind und ggf. einer Modifikation bedürfen.
2. *Vorerfahrungen mit Vorbehandlungen ernst nehmen*
  Wenn Patienten ungünstige Behandlungsvorerfahrungen haben (z. B. starke Nebenwirkungen), wird dies die neue Behandlung in jedem Fall beeinflussen, auch wenn Sie ein neues Präparat wählen. Umso mehr sollte man Vorerfahrungen wertschätzen und immer wieder diskriminative Wahrnehmungen suchen, warum es mit den neuen Behandlungen besser läuft. Ausgesprochen negative Vorerfahrungen könnten auch ein Grund dafür sein, ganz auf eine medikamentöse Behandlung zu verzichten.
3. *„Shared decision making“*
  Beteiligen Sie Patienten an der Behandlungsentscheidung so stark wie möglich.
4. *„Informed consent“*
  Bemühen Sie sich um eine balancierte und vollumfängliche Aufklärung, die nicht nur die Negativeffekte darstellt, sondern immer auch die erwarteten Positiveffekte, die Sie zu dieser Behandlungsentscheidung motivieren.
5. *Therapeutische Beziehung*
  Bemühen Sie sich um eine Wahrnehmung Ihrer Person als kompetent und warmherzig/emphatisch. Dies bahnt, dass Patienten weniger Nebenwirkungen erleben, beim Erleben von Nebenwirkungen eher bereit sind, diese zu akzeptieren und auch sonstige Informationen von Ihnen ernsthafter aufnehmen.
6. *Machen Sie eine personalisierte Kosten-Nutzen-Abwägung*
  Viele positive Effekte, die man mit Psychopharmaka verbindet, sind auch über andere Interventionen erreichbar. Gleichzeitig ist insbesondere für Kurzzeiteffekte ein spezifischer Vorteil von Antidepressiva gut belegt, der auch genutzt werden sollte, wenn die weiteren Bedingungen für eine solche Behandlung günstig sind. Umso mehr: Planen Sie auch eine langfristige Strategie und informieren Sie vor Behandlungsbeginn darüber, ob diese mit einer dauerhaften (ggf. lebenslangen) Medikation einhergeht oder wie und wann ein Absetzen geplant ist.

### Infobox 2 Ein kleines Glossar

*„Balanced placebo design“*: Um Erwartungseffekte und Medikamenteneffekte wirklich trennen zu können, sind Studiendesigns notwendig, die unabhängig sowohl die Medikamentenapplikation (z. B. Verum vs. Placebo) als auch die Erwartungen (z. B. Instruktion, Medikament zu erhalten vs. Instruktion, kein wirkungsvolles Medikament zu erhalten) variieren.

*Placeboeffekt, „placebo effect“*: Veränderungen, die direkt auf Placebomechanismen zurück gehen (und nicht z. B. auf episodische Krankheitsverläufe).

*Placebomechanismen:* Neurobiologische und psychologische Mechanismen, die zum Placeboeffekt beitragen. Psychologisch sind dies z. B. die Rolle von Erwartungen, Informationen und Behandlungskontext, die therapeutische Beziehung oder Lernprozesse/Vorerfahrungen. Neurobiologische Mechanismen können sowohl zentralnervöse Regulationsprozesse sein als auch periphere neurobiologische Prozesse. Placebomechanismen können nicht nur bei Placebobehandlungen, sondern auch bei aktiven Behandlungen auftreten.

*Placeboresponse*: Ausmaß der (meist positiven) Reaktion eines Patienten, wenn er/sie eine Placebobehandlung erfährt.

## Fazit für die Praxis

- Die Wirkung eines Antidepressivums ist nicht allein spezifisch durch die Pharmakodynamik des Präparats, sondern durch zahlreiche weitere Faktoren (mit-)beeinflusst: Die Optimierung von Patientenerwartungen an die Behandlung kann die Behandlungseffektivität steigern. Eine starke therapeutische Beziehung, die durch die Wahrnehmung von Kompetenz und Wärme auf Seiten des Behandlers geprägt ist, kann insbesondere Negativeffekte reduzieren.
- Weit über 90 % der Medikamentenstudien führen keine Analyse von Langzeiteffekten durch. Entsprechend gering belastbar sind unsere Erkenntnisse zu möglichen Vorteilen einer Langzeitbehandlung mit Antidepressiva. Da es sich bei Depressionen um eine biopsychosoziale Erkrankung handelt, müssten Medikamentenstudien noch mehr untersuchen, ob und wann in Interaktion mit welchen Umgebungsbedingungen Antidepressiva hilfreich sind und bessere Effekte haben.
- Studiendesigns zur Evaluation von Antidepressiva sind gefordert, die die komplexen Wechselwirkungen zwischen der eigentlichen Medikamentenwirkung und Placeboeffekten besser darstellen.
